# Improved metabolic health among the obese in six population surveys 1986 to 2009: the Northern Sweden MONICA study

**DOI:** 10.1186/s40608-015-0040-x

**Published:** 2015-02-22

**Authors:** Martin Benckert, Mikael Lilja, Stefan Söderberg, Mats Eliasson

**Affiliations:** Department of Public Health and Clinical Medicine, Sunderby Research Unit, Umeå University, Umeå, Sweden; Department of Public Health and Clinical Medicine, Östersund Research Unit, Umeå University, Umeå, Sweden; Department of Public Health and Clinical Medicine, Umeå University, Umeå, Sweden

**Keywords:** Obesity, Overweight, Time trends, Cohort, Metabolism, Cholesterol, Blood pressure, Diabetes, Physical activity

## Abstract

**Background:**

The incidence of CVD is decreasing in spite of increasing BMI in the population. We examined trends in metabolic health among overweight and obese individuals and the influence of lifestyle and socioeconomic status. Six cross sectional population surveys in the Northern Sweden MONICA Study between 1986 and 2009. 8 874 subjects 25 to 64 years participated (74% participation rate). Metabolic health was defined as a total cholesterol level below 5.0 mmol/l, blood pressure below 140/90 mmHg and not having diabetes. In 2009 the age span 25 to 74 years was studied.

**Results:**

The prevalence of metabolic health among obese subjects increased by 7.9 % per year (95% confidence interval 5.4; 10.5), reaching 21.0% in 2009. The corresponding figures for overweight subjects were 5.9% per year (4.6; 7.3), reaching 18% in 2009, whereas for the normal-weight subjects, the increase was 6.2% per year (5.3; 7.2), reaching 39% in 2009. The prevalence of metabolic health among subjects with abdominal obesity increased by 5.8% (4.6; 7.0) per year, reaching 17.3% in 2009. Among those with no abdominal obesity the increase was 6.2% (5.2; 7.1), reaching 38% in 2009 (*p = <0.001* for all groups). Only among non-obese men and obese women did the increase continue between 2004 and 2009. In the other groups a slight decline or levelling off was noted.

In 2009 women had a 27% higher prevalence of metabolic health than men. The prevalence of metabolic health among the obese was 19.8% which declined to 15.8% if subjects treated for hypertension or hypercholesterolemia were classified as not healthy. Overweight and obese subjects were less often metabolically healthy (odds ratio 0.54 and 0.59 respectively) compared with normal-weight subjects, independent of sex and age as were subjects with abdominal obesity (odds ratio 0.52). Adjustments for smoking, physical activity and education level did not influence any estimates.

**Conclusions:**

This report shows a large increase in prevalence of metabolic health from 1986 to 2009 for all anthropometric categories. Metabolic health remains considerably less prevalent among overweight and obese subjects than among those with normal weight.

## Background

While the number of obese and overweight people continuously increases, the incidence of cardiovascular disease (CVD) as a whole is decreasing [1-5]. In Sweden, all CVD risk factors, exempting obesity [6] and diabetes are decreasing [7]. Recent investigations into the obese and overweight population of Sweden have shown that overweight does not lead to increased mortality [8,9], and a recent meta-analysis with a sample of almost 3 billion people extended these findings to subjects with BMI 30–35 as well [10]. However, this has been contradicted in other studies, which have shown an increased mortality for obese individuals [11-13].

It has been suggested that within the obese population there is a subgroup of subjects that lack the clustering of risk factors seen in the obese population in general, and these “healthy obese” will have less risk for CVD [14-16] although a recent systematic review disputed this [17]. Increased cardiovascular fitness (“fat but fit”) and differences in socioeconomic factors, such as living conditions and education levels, have been suggested as explanations [18-21]. It is also possible that a previously high level of CVD risk factors among the obese has decreased in parallel with, or even more so than, the decrease in the general population. No repeated cross-sectional population based studies has been published, to the best of our knowledge.

In this paper we report time trends over 23 years in the population of northern Sweden in the prevalence of metabolic health in obese and overweight versus normal-weight individuals, as well as in people with or without abdominal obesity. Further, we explore if physical activity, smoking and education modify the effect of obesity on metabolic cardiovascular risk factors.

## Methods

The Northern Sweden MONICA study conducted six population based surveys between 1986 and 2009 [7]. For each survey 250 men and 250 women from each 10-year age group were randomly sampled from population registers. For the time trend analysis we used ages 25–64 years and for the analysis of predictors in 2009 we used 25–74 years. Details on sampling, selection and measurement methods [22], and data on non-participants [7] have been described previously.

The procedures were standardized across surveys. Subjects wore light clothes and no shoes. Weight was measured to the nearest 0.2 kg, and height to the nearest cm [3]. Waist circumference was measured midway between the lower rib margin and the iliac crest. Until 1994, a daily-calibrated balance scale was used, and from 1999 and onwards, an electronic scale was used.

Regarding physical activity, subjects were divided into two groups based on whether they performed at most two light walks per month or more frequent physical exercise. Subjects were divided by educational level into two groups based on whether they had any university level education or at most, secondary or vocational school education. Subjects who answered yes to the question “Do you have diabetes mellitus?” in the questionnaire were classified as having “known diabetes”.

Normal weight was defined as body mass index (BM)I < 25, overweight as BMI between 25 and 30, and obesity as BMI > 30. Abdominal obesity was defined as a waist circumference > 94 cm for men and >80 cm for women.

A metabolically healthy individual, designated also as having metabolic health, was defined as not having hypertension or hypercholesterolemia, using cut-offs proposed by the Fifth Joint Task Force of the European Society of Cardiology and other experts on cardiovascular disease prevention in clinical practice [23]. These levels were blood pressure <140/90 mmHg, total cholesterol <5.0 mmol/l, and no known diabetes, irrespective of treatment with anti-hypertensives or lipid-lowering agents. In an ancillary analysis only subjects without such treatment were classified as metabolically healthy.

MONICA is covered by multiple ethical permissions from The Regional Ethical Review Board, Umeå, Sweden, the latest in 2008. All participants gave written consent.

### Statistical analysis

Statistical significance of differences between anthropometric groups was tested using x^2^-tests where the variables were categorical and ANOVA where the variable were on a continual scale. Generalized linear models and logistic regression were used to adjust for differences in age and sex between groups and to calculate annual percent change. Time trends in the prevalence of metabolic health were analysed by linear-by-linear x^2^-tests. Confidence intervals (CI) are 95%.

## Results

### Trends in metabolic health according to BMI

**Figure 1:**
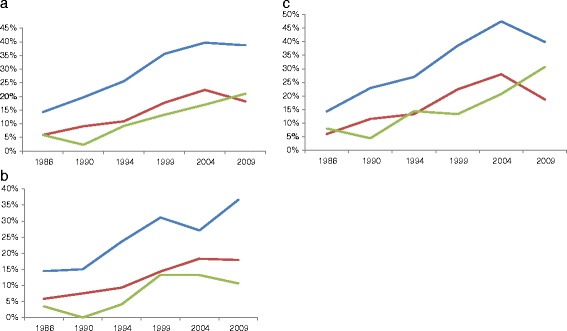
**Time trends in metabolic health for subjects aged 25–64, by BMI-category; a) the whole population; b) men; c) women.** Normal-weight (blue), overweight (red), obese (green).

Altogether 8 874 subjects aged 25 to 64 years participated in the six population surveys 1986 to 2009, 74% of those invited. The prevalence of obese subjects with metabolic health increased by 7.9 % (95%CI 5.4; 10.5) per year, reaching 21.0% (95%CI 17; 26) in 2009. The corresponding increase for overweight subjects were 5.9% (4.6; 7.3) per year, reaching 18% (15; 22) in 2009, and the increase was 6.2% (5.3; 7.2) per year among normal-weight subjects, reaching 39% (35; 43) in 2009 (*p = <*0.001 for each group; Figure 1a). Among normal-weight and overweight subjects a slight decrease in the prevalence of metabolic health was noted between 2004 and 2009. There was no interaction between survey year and BMI category.

In normal-weight men the prevalence of metabolic health increased approximately linearly, but this trend was attenuated for the overweight men and decreased for the obese between 2004 and 2009 (Figure 1b). The overweight women had a lower prevalence of metabolic health than the obese in 1986, 1994 and 2009 (Figure 1c). In normal-weight and obese women, but not overweight women, the prevalence of metabolic health decreased between 2004 and 2009. Time trends were highly significant for all sex and BMI categories (*p < 0.001*).

### Trends in metabolic health according to waist circumference

**Figure 2:**
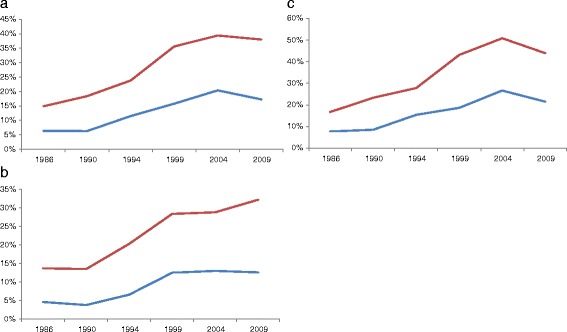
**Time trends in metabolic health for individuals aged 25–64, by abdominal obesity; a) the whole population; b) men; c) women.** Normal-weight (red), abdominal obese (blue).

The prevalence of individuals with abdominal obesity who were metabolically healthy increased by 5.8% (CI 4.6; 7.0) per year, reaching 17.3% (15; 20) in 2009. Among those with no abdominal obesity the increase was 6.2% (CI 5.2; 7.1), reaching 38% (34; 42) in 2009 (Figure 2a). Both in subjects with or without abdominal obesity there was an increase in metabolic health up to 2004, and thereafter a decline. Similar results were found after adjusting for age and gender. There was no interaction between survey year and abdominal obesity.

In men without abdominal obesity, the prevalence of metabolic health increased linearly, while in those with abdominal obesity an initial increase in metabolic health was attenuated after 1999 (Figure 2b). Among women with or without abdominal obesity the prevalence of metabolic health increased, reaching the highest level in 2004, and thereafter metabolic health decreased in 2009 (Figure 2c). Time trends were highly significant for all sex and waist circumference categories (*p < 0.001*).

### Anthropometric predictors of metabolic health in 2009

**Figure 3:**
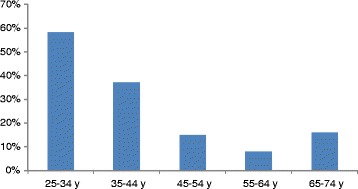
**Prevalence of metabolic health in 10-year age groups in 2009.**

In 2009, 1719 men and women aged 25 to 74 years participated, 69% of those invited. The mean age for the group with metabolic health was 42 years, and it was 54 for those without metabolic health (*p < 0.001*). Women had a 6.5%-units (1.9; 10) higher prevalence of metabolic health than men, 27.9 vs 21.9% (*p =* 0.004). After adjusting for age, the odds ratio for metabolic health was 0.73 (0.58; 0.93), comparing men to women. The prevalence of metabolic health declined with age but increased in the oldest age group (*p = <0.001,* Figure 3).

Roughly one third of the subjects with normal weight had metabolic health while less than one fifth of those with overweight or obesity achieved metabolic health (Table 1). Adjusting for age and sex, the odds ratios for metabolic health were 0.54 (95%CI 0.41; 0.72) and 0.59 (95%CI 0.43; 0.82) for overweight and obese subjects, respectively, compared with normal-weight subjects. Further adjustment for smoking, physical activity and education level did not influence the estimates. Similar proportions and odds ratios were found when comparing those with or without abdominal obesity.

**Table 1:** **Prevalence of metabolic health in 2009 according to degreee of obesity in ages 25 to 74 years**

**Degree of obesity**	**Prevalence (%) of metabolic health (95% CI)**	**Odds ratio (95% CI)**	**Adjusting for age and sex**	**Adjusting for age, sex, smoking, education and physical activity**
**Obesity according to BMI**				
Normal weight	34.6	Reference category	Reference category	Reference category
(n = 694)	(31; 38)			
Overweight	17.5	0.40	0.54	0.52
(n = 651)	(15; 21)	(0.31; 0.52)	(0.41; 0.72)	(0.39; 0.70)
Obesity	19.8	0.47	0.59	0.60
(n = 359)	(16; 24)	(0.34; 0.62)	(0.43; 0.82)	(0.43; 0.84)
**Obesity according to waist circumference**				
No abdominal obesity	35.1	Reference category	Reference category	Reference category
(n = 930)	(32; 39)			
Abdominal obesity	16.5	0.36	0.52	0.52
(n = 774)	(14; 19)	(0.29; 0.46)	(0.41: 0.67)	(0.40; 0.67)

Odds ratios and 95% confidence intervals (CI). *P* values for all comparisons <0.001.

### Influence of lifestyle and socioeconomic status

Altogether 18.8% of the smokers in 2009 had metabolic health, compared with 25.8% of the non-smokers (p = 0.04), a difference of 7.0%-units (0.4; 12). Subjects with a university level education had 7.7%-units (3.1; 12) higher prevalence of metabolic health, 30.4% compared with 22.8% of those with a non-academic education (p = 0.001). These differences were no longer significant after adjusting for age and sex. Of those that exercised once a week or more, 26.6% had metabolic health, compared with 19.5% of those who exercised less than once a week (p = 0.008), a difference of 7.9%-units (2.7; 12). After adjusting for age and sex, exercising less than once a week was associated with 31% less probability of metabolic health (OR 0.69; CI 0.50; 0.92).

#### Influence of treatment with lipid lowering agents or anti-hypertensives

Over the whole period 1986 to 2009, 10.0% of participants, 25 to 64 years, were treated with anti-hypertensives (n = 776), lipid lowering drugs (n = 218) or both. In 2009, 23.7% of participants, 25 to 74 years, were treated with anti-hypertensives (n = 321), lipid lowering drugs (n = 208) or both. Applying the previous definition of metabolic health but categorizing drug treated subjects as not metabolically healthy, irrespective of their blood pressure and cholesterol levels, all analyses were repeated. In general, all prevalence estimates were 2-5%-units lower. In 2009, the prevalence of metabolic health among women 25 to 74 years thus decreased from 27.9% to 24.8% and among men from 21.9% to 17.0%. The proportion metabolically healthy obese in 2009 using this definition was 15.8% compared to 19.8% with the original definition, corresponding estimates among overweight subjects were 13.1% vs 17.5% and among normal weight subjects 31.4 vs 34.6%.

## Discussion

We found a large improvement in metabolic health (defined as having a normal blood pressure, normal cholesterol and no known diabetes) between 1986 and 2009 in all weight categories and in both men and women in the two northernmost counties of Sweden. However, women were generally healthier than men. The increase in metabolic health was attenuated after the turn of the millennium, and the trend was reversed after 2004 for normal- and overweight women and obese and overweight men. Notably we have not found any previous study reporting long-term trends in metabolic health in a well-defined population using a standardized methodology. As recently reviewed [24], there is no consensus on the definition of metabolic health among the obese. As our focus was to explore the impact of obesity on CVD risk we choose to rather use the well- validated risk factors systolic blood pressure, total cholesterol and absence of diabetes. A comparison of prevalence data with other studies is not possible as the 27 studies reviewed used 30 definitions of metabolic health and prevalence ranged between 6% and 75%, with strong age dependence.

The MONICA and NHANES studies [2,7] have shown decreasing CVD risk factors over many years, which are the underlying forces that drive the increase in metabolic health. Could the decreasing metabolic health from 2000 represent a new development and be related to changing diets such as the “low carb, high fat” diet which has received a great deal of attention? Thus, a long-term and very promising trend towards improved metabolic health among the obese seems to have been broken. The recently finished 2014 MONICA survey will possibly provide some answers.

In 2009, CVD risk factors still accumulated in overweight and obese subjects, more so in men than in women. This indicates that while a larger proportion of these subjects now are metabolically healthy, it is still detrimental for one’s health to be overweight and obese, supporting finds from the Framingham and NHANES studies [2,12,25]. However, the larger improvement among the obese may help to explain the paradox of decreasing CVD while obesity increases.

Abdominal obesity was associated with a lower prevalence of metabolic health and higher prevalence of risk factors for CVD. Despite the fact that the subjects with abdominal obesity more than doubled their metabolic health over the 23-year observation period, from 6.4% to 17.3%, it was still half as common for them to be healthy, compared with those without abdominal obesity. Those without abdominal obesity were as metabolically healthy as the normal-weight individuals, and those with abdominal obesity had a similar prevalence of metabolic health as the overweight or obese individuals. As been reported in NHANES and other studies [26-28], abdominal obesity is a better predictor for unhealthiness, but we could not discern any significant difference in clustering of the three risk factors, hypertension, cholesterol and diabetes, between the methods of measuring obesity.

Interestingly, in 2009 the obese women had a greater prevalence of metabolic health than the overweight women, contrary to the expected pattern seen in men. This result may be driven by the higher cholesterol among overweight women compared with the obese, as cholesterol below 5.0 mmol/l was a criterion for metabolic health. However, we have also previously reported that between 2004 and 2009, waist circumference decreased and hip circumference increased among women in northern Sweden [3]. It is noteworthy that a protective effect of larger hip circumference adds considerably to the predictive value of waist circumference on incidence and mortality of CVD as pointed out in a recent systematic review [29].

In 2009, those subjects with metabolic health had a mean age roughly 10 years younger than those without metabolic health. However, even among the youngest, less than 60% had metabolic health. In subjects aged 65 years or more, prevalence of metabolic health slightly increased compared to those 55–64 years of age. The explanation for this is unclear. Perhaps retired persons have more time for exercise and adopt a healthier lifestyle today and ignore the latest trends such as “the low carb, high fat diet”. It is also possible that the health care system more actively diagnoses and treats hypertension and hyperlipidemia in the elderly. Younger individuals may be encouraged to change their lifestyle in lieu of treatment and may not succeed or have little contact with their health care center and therefore risk factors are not assessed.

Smoking was not associated with poorer metabolic health. However, other studies have shown smoking and obesity to have a synergistic effect on cardiovascular risk [25]. With higher education it was more likely for an individual to be of normal weight and metabolically healthy, as is supported by two Swedish studies [9,30], which found that a higher education makes one less likely to be obese and thus more likely to be metabolically healthy. However, adjustment for age and sex abolished the difference between education groups, which supports the idea that life style and primary prevention in Sweden may not bias the socially disadvantaged as much as in other countries.

Subjects with regular physical exercise more often had metabolic health, although less exercise did not explain less metabolic health in obesity or overweight. In a recent study [18] fitness was associated with metabolic health in the obese. Furthermore, fitness was shown to reduce cardiovascular mortality for overweight and obese individuals [21]. Since we had no measure of fitness, we used the self-reported physical activity as a proxy, but still our findings corroborate those fitness studies.

### Strength and limitations

Self-reported weight and height are prone to bias [31], and while questionnaires can provide fairly valid estimates of known diabetes, parameters such as blood pressure, glucose, waist circumference and cholesterol need to be measured. Thus, a valid description and analysis of cardiovascular risk factors, diabetes and their relationship with obesity, on a population level must be based on a physical examination of a random sample, not only on postal questionnaires, which are a common instrument in public health research.

In the Northern Sweden MONICA study, a strict and uniform methodology has been used throughout the whole time period, from 1986 to 2009. Newer methods of analysis were adjusted after re-running older samples, and all anthropometric measurements were performed by trained staff using similar equipment and protocols. This provides a wealth of highly comparable data from an extended time period. Both internal and external validity is high, and it is possible to take common confounders such as socioeconomic status into consideration.

The major limitation of this study is a declining participation rate over the study period. This is most pronounced among the younger population and could present a problem with selection bias. Telephone interviews with the majority of the nonparticipants in the first three surveys (1986, 1990, 1994) showed that they were more likely to smoke (despite similar levels of education) and less likely to be obese or hypertensive than the participants. For the 2009 survey, nonparticipants were younger with lower education and a higher prevalence of diabetes and regular smoking. This may lead to the 2009 data painting an overly optimistic picture, as a higher prevalence of diabetes and smoking should lower the amount of metabolically healthy individuals.

Our definition of metabolic health is arbitrary but based on the most recent European guidelines for cardiovascular prevention, decided upon by all the relevant scientific organizations and systematic reviews [23]. We did not have data on triglyceride or HDL-cholesterol levels, which could have helped to further refine the concept of metabolic health but perhaps not adding much to estimating CVD risk. The variables used were those that form the basis for the cardiovascular risk score (SCORE) proposed by the European Society of Cardiology.

In the definition of metabolic health, treatment for the included risk factors was not taken into account. This could be called into question as treatment of these risk factors probably does not remove all the associated cardiovascular risk. Therefore, we performed an ancillary analysis by classifying treated subjects as not metabolically healthy even if their blood pressure and cholesterol levels were normal. The same general pattern in associations persisted although the absolute levels were lower, most notably among the obese, as expected. This sensitivity analysis strengthens our findings. The dichotomization of reported physical activity may also be a too coarse and blunt instrument to measure and explore a complicated life style such as physical activity.

## Conclusions

While the prevalence of overweight and obesity is increasing in the population of northern Sweden, we can for the first time report that a larger proportion within all weight groups are metabolically healthy over the 23 year observation period. Thus, the strength of obesity as a CVD risk factor may be attenuated [15]. Frequent exercise may contribute to making obesity a more benign condition from a cardiovascular point of view. Surprisingly, being overweight was not substantially different from being obese or having abdominal obesity regarding metabolic health.

As the improvement in metabolic health is slowing down and even reversing for some groups, there is still cause for concern. In addition to traditional cardiovascular risk factors, assessment of cardiorespiratory fitness would be useful to risk stratify individuals with overweight and obesity. A comprehensive analysis of these variables would help in risk prediction and individualized advice and support in primary prevention of CVD and diabetes.

## References

[1] Rosengren A, Eriksson H, Hansson PO, Svardsudd K, Wilhelmsen L, Johansson S (2009). Obesity and trends in cardiovascular risk factors over 40 years in Swedish men aged 50. J Intern Med.

[2] Gregg EW, Cheng YJ, Cadwell BL, Imperatore G, Williams DE, Flegal KM (2005). Secular trends in cardiovascular disease risk factors according to body mass index in US adults. JAMA.

[3] Lilja M, Eliasson M, Stegmayr B, Olsson T, Soderberg S (2008). Trends in obesity and its distribution: data from the Northern Sweden MONICA survey, 1986–2004. Obesity (Silver Spring).

[4] Araujo F, Gouvinhas C, Fontes F, La Vecchia C, Azevedo A, Lunet N (2013). Trends in cardiovascular diseases and cancer mortality in 45 countries from five continents (1980–2010). Eur J Prev Cardiol.

[5] Lundblad D, Holmgren L, Jansson JH, Naslund U, Eliasson M (2008). Gender differences in trends of acute myocardial infarction events: the Northern Sweden MONICA study 1985–2004. BMC Cardiovasc Disord.

[6] Rosengren A, Eriksson H, Larsson B, Svardsudd K, Tibblin G, Welin L (2000). Secular changes in cardiovascular risk factors over 30 years in Swedish men aged 50: the study of men born in 1913, 1923, 1933 and 1943. J Intern Med.

[7] Eriksson M, Holmgren L, Janlert U, Jansson JH, Lundblad D, Stegmayr B (2011). Large improvements in major cardiovascular risk factors in the population of northern Sweden: the MONICA study 1986–2009. J Intern Med.

[8] Ringback Weitoft G, Eliasson M, Rosen M (2008). Underweight, overweight and obesity as risk factors for mortality and hospitalization. Scand J Public Health.

[9] Nyholm M, Merlo J, Rastam L, Lindblad U (2005). Overweight and all-cause mortality in a Swedish rural population: skaraborg hypertension and diabetes project. Scand J Public Health.

[10] Flegal KM, Kit BK, Orpana H, Graubard BI (2013). Association of all-cause mortality with overweight and obesity using standard body mass index categories: a systematic review and meta-analysis. JAMA.

[11] Whitlock G, Lewington S, Sherliker P, Clarke R, Emberson J, Prospective Studies Collaboration (2009). Body-mass index and cause-specific mortality in 900 000 adults: collaborative analyses of 57 prospective studies. Lancet.

[12] Wilson PW, D’Agostino RB, Sullivan L, Parise H, Kannel WB (2002). Overweight and obesity as determinants of cardiovascular risk: the Framingham experience. Arch Intern Med.

[13] Adams KF, Schatzkin A, Harris TB, Kipnis V, Mouw T, Ballard-Barbash R (2006). Overweight, obesity, and mortality in a large prospective cohort of persons 50 to 71 years old. N Engl J Med.

[14] Bluher M (2010). The distinction of metabolically ‘healthy’ from ‘unhealthy’ obese individuals. Curr Opin Lipidol.

[15] Hamer M, Stamatakis E (2012). Metabolically healthy obesity and risk of all-cause and cardiovascular disease mortality. J Clin Endocrinol Metab.

[16] Morkedal B, Vatten LJ, Romundstad PR, Laugsand LE, Janszky I (2014). Risk of myocardial infarction and heart failure among metabolically healthy but obese individuals: HUNT (Nord-Trondelag Health Study). Norway J Am Coll Cardiol.

[17] Kramer CK, Zinman B, Retnakaran R (2013). Are metabolically healthy overweight and obesity benign conditions?: a systematic review and meta-analysis. Ann Intern Med.

[18] Ortega FB, Lee DC, Katzmarzyk PT, Ruiz JR, Sui X, Church TS (2013). The intriguing metabolically healthy but obese phenotype: cardiovascular prognosis and role of fitness. Eur Heart J.

[19] Haapanen-Niemi N, Miilunpalo S, Pasanen M, Vuori I, Oja P, Malmberg J (2000). Body mass index, physical inactivity and low level of physical fitness as determinants of all-cause and cardiovascular disease mortality–16 y follow-up of middle-aged and elderly men and women. Int J Obes Relat Metab Disord.

[20] Kanjilal S, Gregg EW, Cheng YJ, Zhang P, Nelson DE, Mensah G (2006). Socioeconomic status and trends in disparities in 4 major risk factors for cardiovascular disease among US adults, 1971–2002. Arch Intern Med.

[21] Wei M, Kampert JB, Barlow CE, Nichaman MZ, Gibbons LW, Paffenbarger RS (1999). Relationship between low cardiorespiratory fitness and mortality in normal-weight, overweight, and obese men. JAMA.

[22] Stegmayr B, Lundberg V, Asplund K (2003). The events registration and survey procedures in the Northern Sweden MONICA Project. Scand J Public Health Suppl.

[23] Perk J, De Backer G, Gohlke H, Graham I, Reiner Z, Verschuren WM (2012). European guidelines on cardiovascular disease prevention in clinical practice (version 2012): the fifth joint task force of the European society of cardiology and other societies on cardiovascular disease prevention in clinical practice (constituted by representatives of nine societies and by invited experts). Int J Behav Med.

[24] Rey-Lopez JP, de Rezende LF, Pastor-Valero M, Tess BH (2014). The prevalence of metabolically healthy obesity: a systematic review and critical evaluation of the definitions used. Obes Rev.

[25] Jonsson S, Hedblad B, Engstrom G, Nilsson P, Berglund G, Janzon L (2002). Influence of obesity on cardiovascular risk. Twenty-three-year follow-up of 22,025 men from an urban Swedish population. Int J Obes Relat Metab Disord.

[26] De Schutter A, Lavie CJ, Patel DA, Milani RV (2013). Obesity paradox and the heart: which indicator of obesity best describes this complex relationship?. Curr Opin Clin Nutr Metab Care.

[27] Janssen I, Katzmarzyk PT, Ross R (2002). Body mass index, waist circumference, and health risk: evidence in support of current National Institutes of Health guidelines. Arch Intern Med.

[28] Katzmarzyk PT, Janssen I, Ross R, Church TS, Blair SN (2006). The importance of waist circumference in the definition of metabolic syndrome: prospective analyses of mortality in men. Diabetes Care.

[29] Cameron AJ, Magliano DJ, Soderberg S (2013). A systematic review of the impact of including both waist and hip circumference in risk models for cardiovascular diseases, diabetes and mortality. Obes Rev.

[30] Nyholm M, Gullberg B, Haglund B, Rastam L, Lindblad U (2008). Higher education and more physical activity limit the development of obesity in a Swedish rural population. The skaraborg project. Int J Obes (Lond).

[31] Nyholm M, Gullberg B, Merlo J, Lundqvist-Persson C, Rastam L, Lindblad U (2007). The validity of obesity based on self-reported weight and height: implications for population studies. Obesity (Silver Spring).

